# Rhythmic Physical Activity and Global Cognition in Older Adults with and without Mild Cognitive Impairment: A Systematic Review

**DOI:** 10.3390/ijerph191912230

**Published:** 2022-09-27

**Authors:** Gloria Cecilia Vega-Ávila, Diego Fernando Afanador-Restrepo, Yulieth Rivas-Campo, Patricia Alexandra García-Garro, Fidel Hita-Contreras, María del Carmen Carcelén-Fraile, Yolanda Castellote-Caballero, Agustín Aibar-Almazán

**Affiliations:** 1 Faculty of Distance and Virtual Education, Antonio José Camacho University Institution, Santiago de Cali 760016, Colombia; 2 Faculty of Health Sciences, University Foundation of the Área Andina—Pereira, Pereira 660004, Colombia; 3 Faculty of Human and Social Sciences, University of San Buenaventura—Cali, Santiago de Cali 760016, Colombia; 4 Department of Health Sciences, Faculty of Health Sciences, University of Jaén, 23071 Jaén, Spain

**Keywords:** physical exercise, rhythmic, dance, elderly, cognition, randomized controlled trials

## Abstract

Growing evidence suggests that rhythmic physical activity (PA) improves cognitive function in both persons with normal brain aging and with cognitive impairment. This study aims to conduct a systematic review of randomized controlled trials assessing the effects of rhythmic PA over global cognition in older adults with and without mild cognitive impairment. Different keywords related to the topic and Boolean operators were used in the Web of Science, PubMed, and Scopus databases. A total of 11 articles that met the inclusion criteria were analyzed; all of them assessed global cognition using either the Mini-Mental State Examination (MMSE), the Montreal Cognitive Assessment (MoCA) or the Repeatable Battery for the Assessment of Neuropsychological Status (RBANS). Five studies showed beneficial effects over global cognition. All studies had at least one experimental group with rhythmic training, and the interventions evidenced a great diversity of rhythmic stimuli, as well as a varied frequency, duration and type of activities. The heterogeneity of the protocols could be the reason for the mixed findings. Future studies with more precise exercise prescriptions are needed to establish whether rhythmic PA has beneficial effects on global cognition.

## 1. Introduction

The population is undergoing a growth in life expectancy to older ages [1], leading to a worldwide increase in older people [2] and extreme old age [3]. This demographic change justifies the increased attention and concern for the health and well-being of the older adult population, mainly because greater longevity may not be accompanied by a longer period of good health; reports about the situation of older adults who live in developing countries show a slower increase in healthy life expectancy [4]. With the aging process, cognitive changes are evident which may affect the daily function and quality of life of an older adult [5]. Cognitive impairment is considered a public health problem in developing countries due to its high prevalence [6], impact on quality of life [7] and socioeconomic burden [8]. It has been estimated that the annual conversion rate from normal cognition to mild cognitive impairment (MCI) in older adults is 5%, however, this could increase up to 30% when there is suspicion of some type of unidentifiable cognitive impairment in people with a diagnosis of normal cognition [9]. In addition, MCI represents a risk factor for the development of dementias, which are equally prevalent and disabling worldwide [10,11].

Growing scientific evidence suggests that different interventions and exercise modalities have benefits on the health and well-being of older adults, improving physical capacities, metabolic variables, depression, anxiety as well as cognitive function, both in normal states of brain aging and in different stages of cognitive impairment [12,13,14,15,16,17,18,19,20,21]. In particular, it has been suggested that although routine exercise generates physiological brain adaptations, when instructional methods challenge the ability to think and promote different movements, cognitive function is improved and maintained more efficiently [22]; this is typical of interventions based on dance and choreographic activities involving balance and coordination, which have been associated with positive effects on cognition [23], specifically by improving functional connectivity, cognitive performance and increasing brain volumes in the elderly [24]. Rhythmic physical activity (PA) involves a set of varied motor activities that challenge various motor skills, the sense of rhythm, as well as executive functions, in addition to contextual factors that challenge cognitive and emotional control and social skills [25].

Although the mechanisms by which rhythmic PA affects the brain [26], improving its cognitive function, have not been fully understood, it has been identified that the premotor cortex is involved in the auditory rhythm perception [27], as well as in the modulation of cognitive functions, including the understanding of actions, the perception of space and imitation [28]. Likewise, the basal ganglia (BG) are known for detecting the metrical structure of rhythm (or “beat”) [29] and modulating the ability to reproduce rhythm [30], besides, the BG were recently reported to be involved in the regulation of some cognitive functions such as reinforcement learning, decision making, speech fluency, cognition, attention and behavior [31].

During the last decade, several reviews [24,32] have shown that practicing dance is associated with improvements in cognitive function in older adults. However, in these reviews, dance has been accompanied exclusively with rhythmic auditory stimuli, leaving aside other types of stimuli such as visual [33] or tactile stimuli, which also generate perceptions of rhythm [34]. Therefore, the aim of the present study was to conduct a systematic review of randomized controlled trials that evaluated the effects of rhythmic PA or training on cognitive function (global cognition) in older adults with mild cognitive impairment.

## 2. Materials and Methods

The present study corresponds to a systematic review that allowed us to collect evidence about the effect of rhythmic PA on global cognition in older adults. The study was conducted under the guidelines of the PRISMA 2020 document [35,36], and the pre-specified protocol registered in PROSPERO (CRD42022348524).

### 2.1. Sources of Information

Data collection was carried out in June and July 2022 using the following electronic databases: MEDLINE PubMed, Web of Science and Scopus.

### 2.2. Search Strategy

Different keywords were used, as well as the Boolean operators “AND” and “OR”, resulting in the following search string: (“rhythmic task” OR “music-based” OR “rhythmic PA” OR “music exercise training” OR “dance-movement intervention” OR “dance physical training” OR “square dance” OR “tango” OR “aerobic dance” OR “contemporary dance” OR “dance therapy” OR “dancing”) AND (“cognition” OR “cognitive function” OR “cognitive performance” OR “motor-cognitive function” OR “executive function”) AND (“older adults” OR “older women” OR “older men” OR “elderly” OR “seniors” OR “normal cognition” OR “without cognitive impairment” OR “mild cognitive impairment” OR “aging” OR “successful aging” OR “elderly people”).

### 2.3. Inclusion Criteria

The included articles met the following criteria: (1) have at least one intervention group with exercise or rhythmic physical training; (2) include global cognition as an outcome variable; (3) have older adults with mild cognitive impairment or those without cognitive impairment or both, as the study population and age greater than or equal to 50 years; articles in any language.

### 2.4. Exclusion Criteria

We excluded the studies that did not have a control group, showed no comparison results between the rhythmic physical training and the control group, as well as those that did not report the level of global cognition.

### 2.5. Study Selection Process

The selection of articles was carried out using the virtual tool Rayyan [37] (https://rayyan.qcri.org/welcome, accessed on 5 July 2022), which allowed us to discard duplicate articles, to subsequently proceed with the reading of the title and abstract, selecting the articles that met the inclusion criteria; for this purpose, two independent authors (D.F.A.R. and G.C.V.-A.) gave their blinded verdict and in case of disagreement, a third author (P.A.G.-G.) defined the inclusion or non-inclusion of the study.

### 2.6. Data Extraction

The main variable of this review is global cognition. We included data of the authors, year of publication, country of publication, characteristics of the population, (age and presence or absence of cognitive impairment), characteristics of the intervention (type, frequency, intensity, and follow-up time) as well as the results obtained.

### 2.7. Assessment of Methodological Quality

For the assessment of methodological quality, the PEDro scale [38] was used. This instrument consists of an 11-item checklist, which has a maximum score of 10 points, as the first item (“eligibility criteria”) is not used in the final score calculation, each item can be answered as “Yes” (1 point) or “No” (0 points); a score between 0 and 3 was considered “Poor” quality; between 4–5 “Fair”, 6–8 “Good” and >9 “Excellent”. The PEDro website [39] was consulted, collecting the scores of the articles registered there. If no scores were found, two of the authors (G.C.V.-A. and P.A.G.-G.), independently and blinded, manually assessed the methodological quality. Finally, in case of discrepancies between the scores, a third author (D.F.A.-R.) resolved them.

## 3. Results

### 3.1. Selection of the Studies

**Figure 1:**
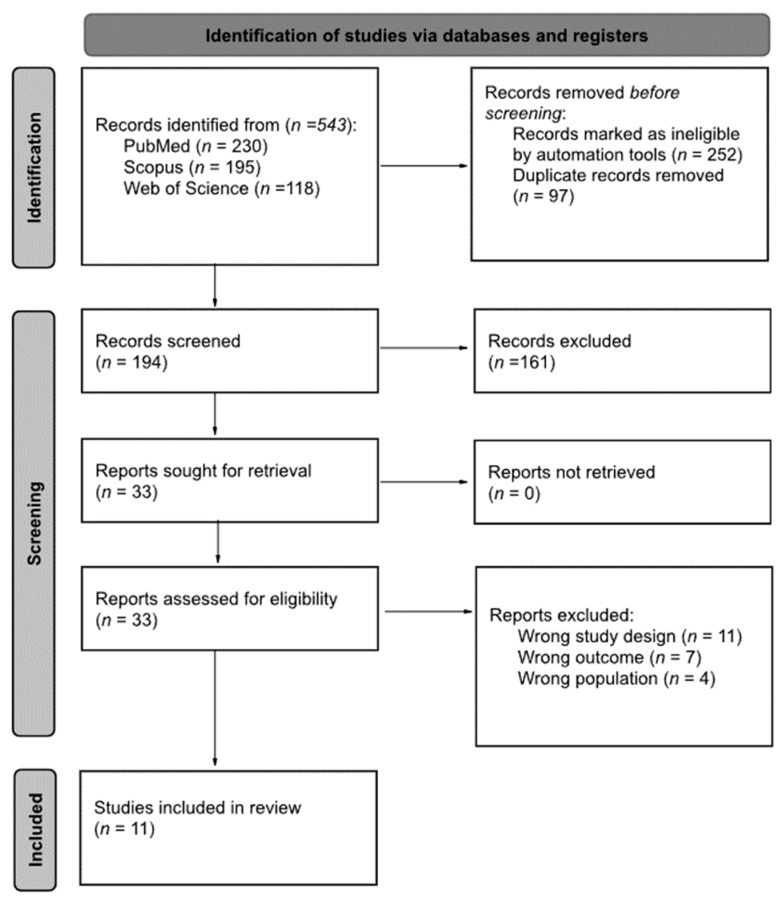
Flow diagram of the study selection process.

The raw search of the databases yielded a total of 543 articles, then an initial filtering within the same databases by document type (Article; Clinical Trial; Randomized Control Trial) and species (Humans) and a subsequent filtering of duplicate articles, left 194 unique articles. These 194 articles were subjected to a review of titles and abstracts, leaving 33 articles as candidates for assessment for eligibility. Only 11 articles met the inclusion criteria [40,41,42,43,44,45,46,47,48,49,50], with 22 articles being excluded (Figure 1).

### 3.2. Methodological Quality

The PEDro scale was used for the evaluation of methodological quality, the score of eight of the articles [40,41,42,43,45,46,47,48] were obtained from the PEDro website, the remaining three articles that were not found on the website were calculated manually [45,50,51], most of the articles were of “good” quality, with scores equal to or higher than 6/10 [40,41,43,44,45,46,48,50], two of the articles were scored as “fair” [42,49] and one as “poor” [47]. The mean score was 5.82 ± 1.47. None of the selected articles blinded the therapists or study participants, one of the articles did not blind the evaluators or present comparisons between groups [47], in one of the articles the eligibility criteria were not presented [46] and one of the articles was not randomized [49]. Table 1 shows the PEDro scale evaluation scores.

### 3.3. Characteristics of the Studies

The articles included in this systematic review were mostly randomized controlled trials, only one was a quasi-experimental, two-group, repeated-measures preintervention, postintervention and 3-month postintervention study [49]. The studies were carried out in Germany [47], Brazil [45], Canada [48], China [42,43,51], Greece [46], Czech Republic [44], United States [49], Spain [40] and Switzerland [43]. All articles were written in English.

In the analyzed studies, 809 people participated (82.45% women and 17.55% men) with an average age of 71.92 ± 5 years. This review included studies with healthy older adults [43,45,47,48,49] and with mild cognitive impairment [40,41,42,44,46,50] or both [43]. A total of 439 people were assigned to experimental groups with different rhythmic training protocols, 355 to control groups and 15 subjects were assigned to other protocols. Table 2 presents the characteristics of the included studies.

### 3.4. Outcomes

The main outcome of the current review was global cognition, assessed using the Montreal Cognitive Assessment (MoCA) in four of the studies [44,48,49,50], while five studies used both the Mini-Mental State Examination (MMSE) and the MoCA [41,42,43,45,46]. Finally, two of the studies [40,47] used the Repeatable Battery for the Assessment of Neuropsychological Status (RBANS). Within the cognitive area, the studies also reported additional assessments for different domains, such as: (a) Memory [40,41,42,44,46,48]; (b) Attention and/or concentration [42,45,47,48]; (c) Executive functions [40,41,44,45,46,48]; (d) Visuospatial function [41,45,47]; (e) Language [40,46]; and (d) Processing speed [40,41,42,45]. Table 3 shows the different tests used in the articles reviewed for the assessment of cognitive domains.

### 3.5. Study Intervention

Each study included both a control and an experimental group in order to compare the effects of rhythmic training on cognition. Four studies based their interventions on light-moderate intensity choreographed aerobic dances [40,41,42,45], where a variety of stimuli and musical styles were involved; in the case of Bisbe et al. [40] the styles used were salsa, rock, rumba, pop and jive. Zhu et al. [41] and Qi et al. [42] used similar routines that consisted of following a sequence of movements that included knee bending exercises, heel raises, boxing, shoulder movements, kicks, square steps, jumping and rowing. Franco et al. [45] designed a program with rhythmic folk songs, including movements in seated, bipedal or a combination of both positions, with changes in speed (fast or slow), and with different alignments such as circles, as well as the possibility of being performed individually, in pairs or in small groups, and also included the memorization of the lyrics of the songs. Finally, Kropacova et al. [44] proposed a dance movement intervention, with different rhythms (Irish country, African dance, Greek dance and tango), which included choreographies divided into several short learning segments, which were continuously combined.

Moreover, the intervention proposed by Lazarou et al. [46] consisted of an international ballroom dance that included rhythms such as the tango, waltz, Viennese waltz, fox trot, rumba, chachachá, swing, salsa, merengue, disco-hustle and traditional Greek ballroom dance, which required different skills such as balance, postural control, dance and rhythm recognition, initiation and completion of movements, turns and displacement in proximity to another individual. Likewise, it was found that in two interventions a program with a specific type of rhythm was implemented; Hackney et al. [49] employed the tango and Chang et al. [50] used Chinese square dance. Meanwhile, Hars et al. [43] included walking sequences following piano music, responding directly or inversely to changes in rhythmic patterns and with changes of direction, as well as exercises for balance control, multidirectional balances, quick reactions exercises and turns. Finally, in relation to this aspect, it was found that in two investigations few details were reported about the structure of the dance-based program [43]. Finally, two studies did not include a detailed description of the design of the dance-based program that they used [47,48]. While Kattenstroth et al. merely mentioned that their intervention followed the Agilando™ guidelines, Esmail et al. [48] only reported that no specific music beat was imposed.

Regarding frequency, the interventions described were mostly performed three times a week [42,43,45,49] or twice a week [41,46,47]; two of the studies presented interventions once a week [43,47] while only one study intervention was performed four times a week [49].

Most of the studies reported that the duration of the intervention period was three months [40,41,42,45,48,49], one study reported a ten months intervention [46] while three reported a six months intervention [44,45,48]. The majority of the articles reported that the duration of each training session was one hour [41,44,45,48,49,51], although times of twenty minutes [45], thirty five minutes [41,42] and a maximum of ninety minutes [49] were reported.

### 3.6. Study Results

#### 3.6.1. Main Outcome: Global Cognition

Bisbe et al. [40] reported intra-group (mean difference 0.24, CI: −0.83, 1.30, *p* = 0.647) and between-group (mean difference 0.23, CI: −0.39, 0.84, *p* = 0.896) differences in global cognition measured with RBANS, however, these were not statistically significant. Likewise, Zhu et al. [41] reported that the intervention used had no effect on the MMSE score, which was modified only by age, where an increase in age was associated with a decrease in MMSE score (*β* IC 95%: −0.366, −0.151–−0.034, *p* = 0.002). In the study of Franco et al. [45] they observed an improvement in physical function but not in cognitive function after the intervention. This absence of improvement in cognitive condition is also evident in the research developed by Hackney [49], who failed to establish the tango as an effective intervention for the variable studied.

According to Esmail et al. [48], there was no effect of time (*p* = 0.92), or group difference or interaction for MoCA (*p* = 0.31). Similarly, Kropacova et al. [44] showed that there were no statistically significant changes for MoCA scores between groups at baseline and at the end of the intervention (*p* = 0.113).

On the other hand, the findings of Qi et al. [42] supported the hypothesis that an intervention with rhythmic PA can effectively improve cognitive function in older adults with MCI (within-group differences demonstrated); in their study, they found that global cognition measured through the MMSE improved significantly in the intervention group (mean ± SD pre: 27. 3 ± 1.3; post: 28.2 ± 1.0; changes = 0.9 ± 1.2; *p* = 0.006) in contrast to the control group where there was hardly any change (mean ± SD pre: 27.1 ± 1.2; post: 27.3 ± 1.7; changes = 0.2 ± 2.1; *p* = 0.730).

The study conducted by Hars et al. [43] evidenced improvement in MMSE score (within-group differences: mean ± SD, from 25.9 ± 2.7 to 26.9 ± 2.1; *t*-test, *p* = 0.004) with an intervention of structured music-based multi-task exercise classes. Lazarou et al. [46] evidenced generalized improvement in cognition and attention (ASD, MMSE and MoCA) after the international ballroom dancing intervention, while for the control group no differences were found (significant differences between groups, *p* < 0.001).

Kattenstroth et al. [47] found beneficial effects of the Agilando™ dance intervention for cognition (within-group pre 0.64 ± 0.02 and post 0.72 ± 0.02, *p* ≤ 0.001) measured with RBANS, evidencing that the intervention was more effective for those who presented a lower baseline physical condition. Additionally, benefits were found in other domains such as posture, reaction, tactile and motor performance and subjective well-being.

Finally, Chang et al. [50] provided evidence for the effects of square dance on cognition improvement, describing significant differences within-group in MoCA after week 9 of intervention (*t* = 4.267, *p* < 0.001), and after week 18 (*t* = 3.400, *p* = 0.001, *d* = 0.71).

#### 3.6.2. Secondary Outcomes: Cognitive Domains

In the studies included in this review, in addition to global cognition, different specific domains of cognition were analyzed through different instruments (see Table 3). In six of the 11 articles reviewed, the analysis of different dimensions of memory capacity were included; the research by Bisbe et al. 2020 [40] showed that the IG, who performed three months of low-moderate intensity choreographed aerobic dance, obtained greater statistically significant benefits in verbal recognition memory from WMS-III compared to the CG (mean difference CI 95%: 1.03, 0.15–1.91, *p* = 0.003) the within-group independent comparison (follow-up from baseline to the end of the intervention), showed a statistically significant improvement in verbal recognition memory from WMS-III in the IG (mean difference 95% CI: 2. 06, 0.79–3.32, *p* = 0.003), in addition, both IG and CG groups significantly improved performance in the visual delayed recall (IG: 95% CI mean difference: 2.29, 0.38–4.21, *p* = 0.022; CG: 95% CI mean difference: 1.57, 0.18–2.96, *p* = 0.030). For their part, Zhu et al. 2022 [41] showed that the intervention with rhythmic PA was associated with episodic memory, i.e., high WMS-RLM scores were obtained in MI (*β* 95% CI: 0.326, 1.005–6.773, *p* = 0.009). Likewise, Kropacova et al. [44] reported that as a result of a 6-month intervention there was an improvement in memory-related dimensions (TCF 1, TCF 2 y WMS III: LogPam2). The above results were aligned with those reported by Qi et al. [42] who demonstrated that WMS-R LM scores increased significantly after their intervention (*p* < 0.05). In contrast, Lazarou et al. [46] reported that their 10-month intervention did not generate significant improvement in memory (RBMT recall, *p* = 0.061). Regarding dimensions related to attention and/or concentration, according to Zhu et al. [41] there was no correlation between intervention with rhythmic PA and attention (DST: *β* 95% CI: 0.154, −1.728–7.217], *p* = 0.225). Nevertheless, Lazarou et al. [46] reported a generalized improvement of attention (TEA) after the international ballroom dancing intervention, while for the CG no differences were found (significant between-group differences, *p* < 0.001). These results were similar to those reported by Kattenstroth et al. [47] (FAIR errors and signs *p* = 0.043 y *p* = 0.008, respectively).

In relation to executive function, heterogeneous results were reported in the reviewed articles. According to Bisbe et al. [40] and Lazarou et al. [46], when comparing the effects of the interventions administered to the IG and CG, no statistically significant differences were observed between the groups on executive function assessed with TMT B [40] and FUCAS [46], nor were intra-group changes evidenced when comparing them before and after the intervention [40,46]. These results were similar to those reported by Hackney et al. [49]. In contrast, Zhu et al. [41] found a correlation between intervention with rhythmic PA and better performance in executive function (TMT B: *β* IC 95%: −0.248, −62.506–−0.278, *p* = 0.048). Similarly, Kropacova et al. [44] and Hars et al. [43] reported that as a result of a 6-month rhythmic PA intervention, executive function improved (Kropacova et al. [44]: ToH 3, ToH 4 and FPT; Hars et al. [43]: FAB, adjusted mean difference between groups 95% CI: 0.12, 0.00–0.25, *p* = 0.047). Finally, Esmail et al. [48] suggested that the improvement in executive function was not specific to the training groups.

On the other hand, according to Bisbe et al. [40], visuospatial function (JLO) showed neither significant differences between groups, nor significant intra-group changes, after two weeks of intervention. In contrast, Kropacova et al. [44] and Lazarou et al. [46] reported significant improvement of visuospatial function after their respective interventions (Kropacova et al. [44]: TCF 1 (*t* (48) = −2.68, *p* = 0.010 y TCF 2 (*t* (48) = −3.48, *p* = 0.001; Lazarou et al. [46]: ROCF delay recall, *p* =0.004). In relation to language, Bisbe et al. [40] reported that verbal fluency (VFC) improved in CG, showing statistically significant differences when compared to IG (mean difference 95% CI: 0.29, 0.11–1.23; *p* = 0.013). As for visual confrontation naming (BNT) and verbal letter fluency (LVF), there were neither significant differences between groups nor significant intra-group changes. In addition, on TMT A processing speed, the articles included in this review reported the following: Bisbe et al. [40] reported no significant differences between the IG vs. CG, and no significant changes intra-group. Likewise, Zhu et al. [41] showed that there was no correlation between intervention with rhythmic PA and processing speed (SDMT: *β* 95% CI: 0.038, −1.475–1.991, *p* = 0.767; TMT A: *β* 95% CI: −0.159, −18.733–4.204, *p* = 0.210), while Qi et al. [42], in their study, reported that SDMT scores significantly increased at the end of the intervention in the IG (*p* < 0.05), but not in the CG.

Finally, it was found that, although Esmail et al. [48] independently assessed memory (WAIS-4 Digit), as well as attention, visuospatial function and processing speed (all through the WAIS-3), they did not report results related to these abilities according to time or type of intervention.

## 4. Discussion

There is growing scientific evidence indicating the association between cognitive function and quality of life in older adult populations [51,52,53]. It has even been reported that greater severity of cognitive dysfunction is associated with greater negative impact on quality of life in Chinese older adults [54]. This highlights the importance of designing evidence-based interventions, such as PA-based interventions, that have the potential to preserve cognitive function. Based on the above, this systematic review aimed to evaluate the effects of rhythmic PA on global cognition in older adults with mild cognitive impairment and without cognitive impairment, therefore, 11 articles that met the criteria established for inclusion were analyzed [40,41,42,43,44,45,46,47,48,49,50]. This review revealed a heterogeneous effect of this type of intervention on the main variable considered in the study.

The main variable studied in this review was global cognition, whose change due to the exercise intervention was measured through different instruments: MMSE, MoCA and RBANS, all of which are comparable to each other allowing similar conclusions regarding the disease burden [55]. In relation to the methodological quality, we found that it ranged between Fair [42,49] and Good [40,41,44,45,46,48,50], however, one of the articles [47] presented a Poor quality. The fact that none of the articles performed a blinding of the therapists or participants could be explained by the nature of the intervention, since this is a common problem that has been reported in other systematic reviews related to the practice of PA in any of its variations [56,57]. Furthermore, four of the articles [42,43,47,49] did not perform a concealed allocation. These are the main features by which articles tend to report inaccurate effects when compared with other clinical trials that do comply with them [58]. Another common point where the different articles presented a problem was the follow-up; 63.6% did not carry out a correct follow-up, so it is impossible to determine whether the effects of the intervention were maintained in the long-term or not. The literature establishes that those articles that present a better methodological quality tend to generate more robust results [59]. In this systematic review, it became evident that the studies with higher methodological quality tended to report that no significant changes were generated between the groups studied [40,41,45,48]; however, this is not enough reason to disregard the findings of the other studies.

Although one of the objectives of this review was to expand the field of knowledge by including experimental studies that evaluated the effects of rhythmic PA, with or without music, on global cognition in this population, all the interventions analyzed had as a central component stimulus that were mainly auditive, using a variety of musical styles; however, the interventions did not mention details about the structure of the rhythms used. This should have been considered since movement reflects, imitates and predicts some musical characteristics related to rhythm, timbre [60], pitch or frequency ranges [61], therefore, auditory functions and musical characteristics have implications in the design of sound-based interventions [61]. To illustrate this, consider that “key pulses” stimulate the use of various types of movement of different body parts, while spectral flow and percussion stimulate the movement of specific body parts, such as head and hands [60]. In addition, changes in pitch influence movement, proprioceptive awareness and feelings about one’s own body, just as changes in frequency range affect the amplitude of movement, body sensations and emotional state [61]. In addition, musical tempo is associated with beat speed and movement speed [62], which could affect cognitive function, since movement speed has been positively associated with cognition in older adults [63].

Additionally, only five of the reviewed studies reported the intensity level used in the interventions; this lack of detail in describing the control variables of the exercise load generates two important problems: first, it impedes the estimation of the dose or range of doses that achieved a minimum clinically relevant and safe improvement on global cognition and, second, it interferes with the comparison of the results obtained with those of other studies. Previously, exercise dose has been defined in terms of energy expenditure (metabolic equivalent of the task, MET) that results from the combination of intensity, type of exercise, duration and frequency [64]; this strategy has been used to determine the effects of other types of interventions with exercise or PA on cognitive function, estimating 724 MET-min per week as the minimum dose to generate positive changes in cognition, while doses higher than 1200 MET-min per week provided less clear benefits [65].

Another aspect to highlight is that the studies that did not achieve positive effects on global cognition were characterized by having interventions with training frequencies of two–four times/week, with a duration ranging from 12 to 26 weeks. In contrast, the interventions that had positive results on global cognition presented slightly lower frequencies of one–three times/week, but with a longer duration ranging from 13 to 43 weeks; this could be interpreted as a compensatory effect between the frequency and the total duration of the intervention, which could be explained by interactive effects between the intensity, frequency and duration of the training [66]. Additionally, it should be noted that, although this review included studies completed with healthy older adults [43,45,47,48,49] with mild cognitive impairment [40,41,42,44,46,50] or both [43], this cognitive level did not seem to influence the final effects reported in relation to global cognition after the interventions. This is clear because inconsistent results were found in relation to global cognition, both in the studies that evaluated healthy adults and in those that evaluated adults with mild cognitive impairment.

Moreover, regarding the different domains of cognition reported in this review, it was found that most of the interventions showed improvements mainly in memory capacity and executive function, which is consistent with previous studies [67] and could be explained by the brain changes induced by this type of interventions [68].

This systematic review updates the status of the available information on the effects of rhythmic PA on global cognition, in addition to having articles with adequate methodological quality. However, there are some limitations that should be taken into account when interpreting our results, including the methodological heterogeneity of the included studies, which limits the possibility of obtaining specific results related with the effect of rhythmic PA on global cognition in the population studied. In addition, a possible publication bias can be ruled out by having included research that reported a statistically significant relationship between the variables studied, as well as studies that did not report a significance for such a relationship.

## 5. Conclusions

Interventions based on rhythmic PA that are currently being implemented with the aim of improving global cognition in older adults with and without mild cognitive impairment are structured in such a way that the perception of rhythm is stimulated mainly through auditory stimuli. However, the heterogeneity in the intervention protocols, the lack of detail to describe the structural characteristics of the rhythms used and some variables related to the training load, could be the cause of the mixed results regarding the effect of rhythmic PA on global cognition. Therefore, it is necessary to design interventions with greater methodological rigor to facilitate the understanding of these types of interventions and their effects. In this sense, this systematic review identifies and analyzes the fundamental methodological aspects of the design of interventions based on rhythmic PA, providing information that allows decision-making based on scientific evidence and therefore can be used as a guide for the design of this type of intervention.

## Figures and Tables

**Table 1:** Methodological quality of the articles included.

Items		1	2	3	4	5	6	7	8	9	10	11	Total
Autor													
Bisbe et al. 2020 [40]		Y	Y	Y	Y	N	N	Y	Y	Y	Y	Y	8
Zhu et al. 2022 [41]		Y	Y	Y	N	N	N	Y	Y	N	Y	Y	6
Qi et al. 2019 [42]		Y	Y	N	Y	N	N	Y	N	N	Y	Y	5
Hars et al. 2014 [43]		Y	Y	N	Y	N	N	Y	N	Y	Y	Y	6
Kropacova et al. 2019 [44]		Y	Y	Y	Y	N	N	N	Y	N	Y	Y	6
Franco et al. 2020 [45]		Y	Y	Y	Y	N	N	Y	Y	Y	Y	Y	8
Lazarou et al. 2017 [46]		N	Y	Y	Y	N	N	Y	N	N	Y	Y	6
Kattenstroth et al. 2013 [47]		Y	Y	N	Y	N	N	N	N	N	N	Y	3
Esmail et al. 2020 [48]		Y	Y	Y	Y	N	N	Y	N	N	Y	Y	6
Hackney et al. 2015 [49]		Y	N	N	Y	N	N	Y	N	N	Y	Y	4
Chang et al. 2021 [50]		Y	Y	Y	Y	N	N	Y	N	N	Y	Y	6

Items: 1 = eligibility criteria; 2 = random allocation; 3 = concealed allocation; 4 = baseline comparability; 5 = blind subjects; 6 = blind therapists; 7 = blind assessors;8 = adequate follow-up; 9 = intention-to-treat analysis; 10 = between-group comparisons; 11 = point estimates and variability; Y = Yes; N = No. The eligibility criteria item does not contribute to the total score.

**Table 2:** Characteristics of the included studies.

Author (Year of Publication)	Sample Size	Sex (% Female)	Age Mean (SD)	Level of Cognition and Screening Tool	Intervention Group: Type, Duration, Frequency, Intensity	Control Group: Program Carried Out	Intervention Duration and Assessments	Measuring Instrument	Main Results
Bisbe et al. 2020 [40]	*n* = 31; IG = 17; CG = 14	48.39	75.08 ± 5.38	MMSE ≥ 24 **	T: Choreographed aerobic dances D: 60 min F: 2 times/week I: Light to moderate intensity (<6 METS), 2–3 Pts on the Borg scale	Different motor abilities, such as strength, endurance, flexibility, balance, coordination and gait were trained, according to physiotherapeutic common practices	12 weeks T0: Baseline T1: 12 weeks	MMSE	No significant differences were found within-group changes after the intervention. After 12 weeks *p* = 0.647
Zhu et al. 2022 [41]	*n* = 54 IG = 29 CG = 25	75.93	70.66 ± 7.18	MMSE ≥ 25 *	T: Aerobic dance D: 35 min F: 3 times/week I: HRmax of 60–80%	Both the intervention and control groups received a health education program (in the form of a 120-min-long lecture) after inclusion in this study. Follow-up: participants were contacted by telephone every week to remind them about educational program highlights	3 months T0: Baseline T1: 3 months	MMSE MoCA	3 months of aerobic dance improves cognitive function. There was a correlation between the intervention and MoCA, as the intervention group (β [95% CI]: 0.280 [0.159, 2.361], *p* = 0.026). Furthermore, an increase in age was associated with a decrease in MMSE score (β [95% CI]: −0.366 [−0.151, −0.034], *p* = 0.002)
Qi et al. 2019 [42]	*n* = 32 IG = 16 CG = 16	71.88	69.85 ± 7.15	MMSE: 25–30; MoCA ≤ 26 **	T: Aerobic dance D: 35 min F: 3 times/week I: HRmax of 60–80%	Received usual care	3 months T0: Baseline T1: 3 months	MMSE MoCA	Within-group differences demonstrated that the scores of MMSE and MoCA were significantly increased in the EG (*p* < 0.05) compared with the baseline
Hars et al. 2014 [43]	*n* = 134 IG = 66 CG = 68	96.27	IG = 75 ± 8 CG = 76 ± 6	MMSE *	T: Structured music-based multitask exercise classes (Jaques-Dalcroze eurhythmics movement method) D: 60 min. F: 1 time/week. I: not reported	The control group maintained their usual physical and social habits	6 months T0: Baseline T1: 6 months	MMSE	Within-group analysis in MMSE scores indicated an increase in the intervention group from baseline to Month 6 (from 25.9 ± 2.7 to 26.9 ± 2.1; *t*-test, *p* = 0.004)
Kropacova et al. 2019 [44]	*n* = 99 IG = 49 CG = 50	76.77	IG: 69.16 ± 5.36 CG: 68.37 ± 6.10	MoCA < 26 points ***	T: Dance movement intervention. D: 60 min. F: 3 times/week I: not reported	Life as usual	6 months T0: Baseline T1: 6 months	MoCA	No statistically significant changes for MoCA results between groups differences at the baseline (*p* = 0.113)
Franco et al. 2020 [45]	*n* = 71 IG = 35 CG = 36	91.55	69 ± 6.6	MMSE ≥ 24 *	T: Senior dance D: 60 min. F:2 times/week. I: Moderate-level intensity (participants had to breathe a little harder than normal)	1 h single educational class on strategies to prevent falls	3 months T0: Baseline T1: 3 months	MoCA	No significant differences between intervention and control groups at 12-week follow-up in cognitive function measured by MoCA (β [95% CI]: 0.6 [−0.7, 1.8])
Lazarou et al. 2017 [46]	*n* = 129 IG = 66 CG = 63	78.29	66.8 ± 10.1	Stage 3 of the disease according to GDS **	T: International Ballroom Dancing. D: 60 min. F:2 times/week I: not reported	Life as usual	10 months (40 weeks) T0 Baseline T1: 40 weeks	MMSE. MoCA	Significant improvements in MMSE after 10 months of dance intervention whereas no improvements were found for the control group. Significant differences between dance intervention and control groups (*p* < 0.001)
Kattenstroth et al. 2013 [47]	*n* = 35 IG = 25 CG = 10	68.57	68.60 ± 1.45	MMSE: 27 to 30 *	T: Special dance program for seniors (Agilando™). D: 60 min F: 1 times/week I: not reported	Life as usual	6 months T0: Baseline T1: 6 months	RBANS	After 6 months of dance intervention, significant improvements in RBANS within the intervention group (*p* ≤ 0.001), whereas no improvements were found for the control group (*p* = 0.361)
Esmail et al. 2020 [48]	*n* = 41 IG1 = 12 IG2= 15; CG = 14	75.61	67.48 ± 5.37	MMSE > 24 *	T: Dance movement training D: 60 min. F: 3 times/week. I: twice a week 110% of MAP, 1 time a week 70% of MAP	Life as usual	3 months T0: Baseline T1: 3 months	MoCA	There was no time effect (*p* = 0.92), group difference or interaction for the MoCA (*p* = 0.31)
Hackney et al. 2015 [49]	*n* = 74 IG = 62 CG = 12	71.62	IG = 82.3 ± 8.8; CG = 84.1 ± 7.9	MoCA; No history of neurodegenerative Disease *	T: Tango D: 90 min. F: 4 times/week I: not reported	90 min of health education classes, for 12 weeks, 4 times per week (20 sessions)	3 months T0: Baseline T1: 1 week T2: 3 months T3: 6 months	MoCA	There were no significant differences between the groups ((*p* = 0.31))
Chang et al. 2021 [50]	*n* = 109; IG = 62; CG = 47	100	EG: 76.56 ± 3.60CG: 75.94 ± 3.61	MoCa < 26 **	T: Square dance exercise. D: 60 min. F: 3 times/week. I: 100–140 bpm	Life as usual	18 Weeks T0: Baseline T1: 9 weeks T2: 18 weeks	MoCA	There were significant differences for week 9 and 18 in MoCA (*p* < 0.001, *p* = 0.001, respectively), in the control group no significant differences were evident. There were no significant differences between groups, (*p* = 0.096).

IG: Intervention Group; CG: Control Group; MMSE: Mini-Mental State Examination; MoCA: Montreal Cognitive Assessment; GDS: Global Deterioration Scale; RBANS: Repeatable Battery for the Assessment of Neuropsychological Status; T: Type; D: Duration; F: Frequency; I: Intensity; HRmax: Maximum Heart Rate; MAP: Maximal Aerobic Power; Pts: Points; * Without mild cognitive impairment. ** With mild cognitive impairment. *** With and without mild cognitive impairment.

**Table 3:** Tests and cognitive domains assessment.

Cognitive Domain	Tests
Overall cognitive level (global cognition)	Montreal cognitive Assessment (MoCA) [41,42,43,44,45,46,48,50] Mini-Mental State Examination (MMSE) [40,41,42,43,45,46] Repeatable Battery for the Assessment of Neuropsychological Status (RBANS) [40,47]
Memory	Taylor figure test recall 3 min after copy (TCF 1) [44] Taylor figure test recall 30 min after copy (TCF 2) [44] Wechsler Memory Scale third edition: Logical memory subtest from WMS III immediate recall (WMS III: LogPam 1) [44] Wechsler Memory Scale third edition: Logical memory delayed recall after 30 min (WMS III: LogPam 2) [44] Wechsler Memory Scale third edition: Delayed Recall (WMS-III: delayed recall) [40] Wechsler Adult Intelligence Scale fourth edition (WAIS-4 Digit): Span forwards and backwards and Similarities [48] Repeatable Battery for the Assessment of Neuropsychological Status: Delayed Recall (Delayed Recall RBANS) [40] Wechsler memory scale-revised logical memory (WMS-RLM) [41,42] Forward Digit Span Task (DST) Chinese version [41] Rivermead Behavioral Memory Test (RBMT) of direct and deferred recall of history [46] The Brooks Spatial Task (a spatial cognition task involving memory of the placement of numbers on an orally described 4 9 4 matrix) [48]
Attention and/or concentration	Forward Digit Span Task (DST) Chinese version [41] Wechsler Adult Intelligence Scale third edition (WAIS III): symbols Symbol search subtest from WAIS III [44,48] Wechsler Adult Intelligence Scale third edition (WAIS III): Digit span subtest from WAIS III [44,48] The paper-and-pencil non-verbal geriatric concentration test-AKT [47] Frankfurt Attention Inventory (FAIR) [47] Test of Everyday Attention (TEA) [46]
Executive function	Backward Digit Span Task (DST) Chinese version [41] Tower of Hanoi—3 disks (ToH 3) [44] Tower of Hanoi—4 disks (ToH 4) [44] Five-point test (FPT) [44] Trail Making Test part B (TMT B) [41,42,46,47,49,50] The Frontal Assessment Battery (FAB) [43] Functional and Cognitive Assessment Test (FUCAS) [46]
Visuospatial function	Judgment of line orientation test (JLO) [40,44] Taylor figure test copy (TCF copy) [44] The Rey–Osterrieth Complex Figure (ROCF copy and delay recall) [46] Wechsler Adult Intelligence Scale third edition (WAIS-3): Substitution [48]
Language	Letter Verbal Fluency (LVF) [40] Test F-A-S for verbal fluency (FAS) [46] Boston Naming Test (BNT) [40] Category Verbal Fluency (CVF) [40] Verbal Fluency F-A-S test (FAS) [46]
Processing speed	Trail Making Test parts A (TMT A) [41,42,46] Symbol Digit Modalities Test (SDMT) [41,42] Wechsler Adult Intelligence Scale third edition (WAIS-3): Substitution [48]

## Data Availability

All available data can be obtained by contacting the corresponding author.
